# Evaluation of bone density and hand grip strength in the course of drug treatment for osteoporosis: A real-world study

**DOI:** 10.1007/s00132-023-04367-5

**Published:** 2023-04-24

**Authors:** Guido Schröder, Ivonne Hoth, Dirk Flachsmeyer, Mario Dutzke, Julian Ramin Andresen, Reimer Andresen, Hans-Christof Schober

**Affiliations:** 1 Clinic of Orthopedics and Trauma Surgery, Warnow Klinik, Bützow, Germany; 2 Clinic of Orthopedic‑, Trauma- and Restorative Surgery—Charité University Medicine, Campus Benjamin Franklin, Berlin, Germany; 3 Institute of Diagnostic and Interventional Radiology/Neuroradiology, Westküstenklinikum Heide, Academic Teaching Hospital of the Universities of Kiel, Lübeck and Hamburg, Heide, Germany; 4 Clinic of Internal Medicine IV, Klinikum Südstadt Rostock, Academic Teaching Hospital of the University of Rostock, Rostock, Germany

**Keywords:** DXA, Older age, Physical fitness, Vertebral fracture, Osteosarcopenia, DXA, Höheres Lebensalter, Körperliche Leistungsfähigkeit, Wirbelkörperfraktur, Osteosarkopenie

## Abstract

**Background:**

The aim of this clinical investigation was to assess the physical performance in osteoporotic patients undergoing drug treatment (DT) for years by measuring hand grip strength (HGS) and bone mineral density (BMD). A further aim was to detect the time until the occurrence of vertebral fractures (VF) and influencing factors.

**Material and methods:**

The investigation comprised 346 persons (276 women, 70 men) aged on average 66.9 ± 10.7 years with confirmed osteoporosis (OP). Over a mean period of 1384 ± 727 days, OP was assessed every 2 years, including a bone densitometry by dual X‑ray absorptiometry and HGS measurement. In subgroups OP patients were analyzed with and without a bone density (BMD) increase, and with and without VFs.

**Results:**

Under DT, calcium and vitamin D substitution, the median T‑score improved in the entire group from −3.2 to −3.1 standard deviations (SD; *p* = 0.002). HGS was reduced (median) from 26 kg to 24 kg (*p* < 0.001). The median interval until the occurrence of VF was 2652 days (95% confidence interval [CI] 1825.2–3478.8 days) and 1461 days (95% CI 1246.5–1675.5, *p* < 0.001) in those with and without a BMD increase, respectively.

**Discussion:**

Guideline-based DT improves bone density and causes a longer interval without VF. The HGS falls independent of BMD. The association between bone and muscle in patients with a deterioration of the musculoskeletal system is known as osteosarcopenia. Early muscle exercises would be meaningful in this setting.

**Graphic abstract:**

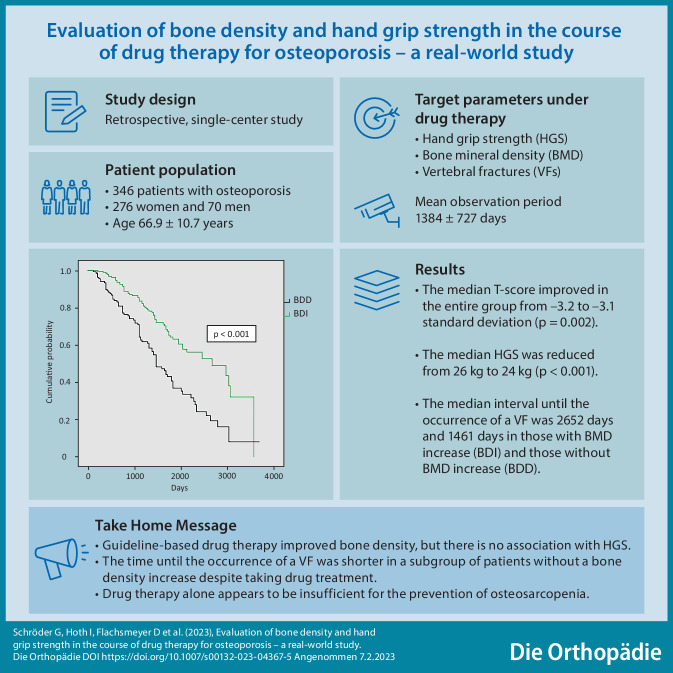

## Abstract (de)

**Hintergrund:**

Das Ziel der vorliegenden klinischen Untersuchung war die Abschätzung der Leistungsfähigkeit des muskuloskeletalen Systems mittels Verlaufsmessung der Knochenmineraldichte („bone mineral density“ [BMD]) und Handgriffkraft (HGS) bei Osteoporose-PatientInnen unter medikamentöser osteologischer Therapie (MOT). Gleichzeitig sollen Aussagen über die Zeit bis zum Auftreten von Wirbelkörperfrakturen (WKF) gemacht und deren Einflussfaktoren evaluiert werden.

**Material und Methoden:**

Insgesamt 346 Personen (276 Frauen/70 Männer) im Durchschnittsalter von 66,9 ± 10,7 Jahren mit gesicherter Osteoporose nahmen an der Untersuchung teil. Über einen Zeitraum von durchschnittlich 1384 ± 727 Tagen erfolgte alle 2 Jahre ein Assessment inklusive Osteodensitometrie mittels „dual x‑ray absorptiometry“ (DEXA, GE Healthcare’s Lunar Prodigy, Aartselaar, Belgien) und einer HGS-Messung (Smedley-Dynamometer, Tokio, Japan). In Subgruppenanalysen wurden Osteoporose-PatientInnen mit und ohne BMD-Anstieg sowie mit und ohne WKF betrachtet.

**Ergebnisse:**

Unter MOT einschließlich Kalzium und Vitamin-D-Substitution kam es im Verlauf zu einer Verbesserung des medianen T‑Score-Werts der Gesamtgruppe von −3,2 auf −3,1 Standardabweichungen (SD; *p* = 0,002). Gleichzeitig verringerte sich die HGS der Gesamtgruppe im Median von 26 auf 24 kg (*p* < 0,001). Die Zeit bis zum Auftreten einer WKF betrug in der Gruppe mit BMD-Anstieg im Median 2652 Tage (95 % Konfidenzintervall [KI] 1825,2–3478,8 Tage) und 1461 Tage in der Gruppe ohne BMD-Anstieg (95 % KI 1246,5–1675,5, *p* < 0,001).

**Schlussfolgerung:**

Die leitliniengerechte MOT führt zu einer Verbesserung der Knochendichtewerte und zu einer längeren WKF-freien Zeit. Unabhängig davon sinkt die HGS. Es gibt einen Zusammenhang zwischen Knochen und Muskulatur bei einer zunehmenden Verschlechterung des muskuloskeletalen Systems – Osteosarkopenie. Ein frühzeitiges Muskeltraining erscheint vor diesem Hintergrund sinnvoll.

## Introduction

Osteoporosis (OP) is a serious problem for patients who experience fractures. In Germany about 8 million persons are affected by OP [20]. The incidence of clinically evident osteoporotic vertebral fractures (VF) is about 1.4 million worldwide [21]. Numerous investigations have shown an association between muscle strength, age, and the risk of fractures [3, 14, 24]. Especially older persons are subject to a higher risk of osteoporotic fractures, which worsen quality of life, cause disabilities, loss of independence, admission to care homes, and higher rates of morbidity and mortality [23, 34]. Furthermore, the fractures lead to a loss of endurance and coordination, which markedly impairs the activities of daily living [31]. A clear pathophysiological association has been observed between OP and sarcopenia, an age-related disease associated with reduced muscle mass, strength and function. The combination of these two diseases is known as osteosarcopenia [34].

Hand grip strength (HGS) has been associated with numerous signs of aging [6] and is a major symptom of sarcopenia [8] and fragility [16]. The HGS in both arms is measured with a hand dynamometer and reflects muscle function in the upper as well as lower extremities [11]. A low HGS proved to be a better predictor of mortality than age and systolic blood pressure [25, 27, 30]. Therefore, the role of HGS as a predictor of healthy aging and a potential instrument of clinical assessment has been a subject of avid interest [7].

The association between the administration of therapeutic agents with different mechanisms of action on areal bone mineral density (BMD), measured by dual X‑ray absorptiometry (DXA), and the occurrence of VFs has been frequently investigated [10]. The majority of these data were registered in studies. Real-world data are rare.

On the other hand, the impact of long-term drug treatment (DT) for osteoporosis on the development of HGS and BMD in the course of disease has been scarcely investigated so far. We addressed this question in the present clinical investigation.

Furthermore, we intended to answer the following research questions:

1. Is the success of treatment defined by the improvement of bone density able to delay the occurrence of VFs?
2. What associations exist between HGS and other clinical parameters in osteoporotic patients?
3. What independent predictors influence the occurrence of VFs?

## Material and methods

### Study design and recruitment

We performed a retrospective single-center clinical investigation of a treatment group. We analyzed subgroups of OP patients with bone mineral density increase (BDI) and with bone density decrease (BDD), as well as those with (VFs) and without VFs (no VFs). Patients were assigned to the BDI and BDD groups according to the improvement of their T‑score on dual X‑ray absorptiometry. All participants were informed comprehensively about the methods, purposes, and risks of the study protocol. They were then asked to sign a written statement of consent to participate in the study. The participants were recruited through the OP outpatient department of the local hospital in their region.

### Inclusion and exclusion criteria

Inclusion criteria for the clinical investigation were the presence of OP requiring treatment in patients with pathological values on DXA (GE Healthcare’s Lunar Prodigy, Aartselaar, Belgium), and the availability of X‑rays of the thoracic (TS) and lumbar spine (LS). At the start of the study, all patients underwent a physical examination to register the orthopedic status in terms of hand grip strength (HGS), the chair rising test (CRT), tandem stance (TDS), and the walking test (WT).

Exclusion criteria were all types of severe heart failure, uncontrolled hypertension, relevant neurological deficits, vestibulopathy, or dependence on a caregiver.

### Declaration of ethics approval

We declare that the present study on human subjects fulfils the Helsinki declaration of 1975, revised in 2000, and was approved by the institutional ethics committee of the university in charge (ethics vote A2020-0041).

### Clinical tests

#### Hand grip strength (HGS)

The HGS was measured in kilograms (kg) using a Smedley‑S dynamometer TMM Tokio 100 kg. In accordance with published recommendations [28], the investigated person assumed a sitting position. The upper arm was adducted and the elbow was flexed at 90°. The forearm and the wrist were in a neutral position. The patient performed three attempts each with the dominant, and then with the non-dominant hand. The mean value was then determined. It should be noted that other studies have yielded similar data, regardless of whether mean or maximum values achieved in several attempts were used [19].

Previous investigations have shown that various types and brands of dynamometers provide similar results; in other words, the reference values are independent of the dynamometer type used [13].

With respect to sarcopenia, men with a value < 27 kg are limited in their performance abilities, whereas the cut-off value for women is 16 kg [9].

#### Chair rising test (CRT)

The CRT in seconds (s) consists of five consecutive acts of standing up from and sitting down in a chair without armrests, with the person’s arms crossed before the chest. The results provide data about a normal or high risk of falls. Scores ≤ 10 s express a normal risk, and scores > 10 s indicate a high risk of falls [29]. The test is also used to assess the presence of sarcopenia [9].

#### Tandem stance (TDS)

The TDS is used to assess balance and coordination. The position is held for 10s. The patient is free to decide which foot remains in the front [29].

#### Walking test (WT)

The walking test measures the time taken to walk a distance of 4m at normal speed. This task combines the patient’s strength and coordination when walking and is therefore a good parameter for the assessment of physical performance. The threshold for limited mobility is a walking speed of ≤ 0.8 m/s [9].

#### Short physical performance battery (SPPB)

The battery consists of three tests: TS, walking over a distance of 4m, and the CR test [17]. For each task the patient can achieve a maximum of 4 points. Finally, the results of all 3 tests are added so that a minimum of 0 and a maximum of 12 points can be achieved. We used the SPPB to measure the function of the lower extremities with tasks similar to the activities of daily living. The total score permits an assessment of the person’s degree of limitation in daily life. A final score of 0–3 points shows that the person is severely impaired, especially when walking a few 100m, when ascending stairs, and in self-care [17]. Patients with scores of 4–6 points have a moderate, and those with 7–9 points a mild limitation of their mobility. If the patient achieves a score of 10–12 points, he/she is either minimally limited or not limited at all in daily life. In general, the SPPB is a frequently used instrument with a proven ability to identify and characterize persons at the disabled end of the functional spectrum, as well as older persons with no disabilities [18].

### Pain

In the present investigation we used the numeric rating scale (NRS). This is a unidimensional pain scale consisting of 11 grades: 0 expresses no pain and 10 stands for the worst pain imaginable. Within this range, the probands select the number that best expresses their perception of pain. The advantages of the NRS include a low error rate of results and a high degree of acceptance by test subjects [2].

### Bone densitometry

The DXA is the most frequently used procedure to measure bone density in OP. The WHO defines it as the standard method for a metrological definition of OP [1].

Bone densitometry of the hips comprises four regions: the femoral neck, the trochanter region, the intertrochanter region, and Ward’s triangle. At the axial skeleton one mainly measures bone density at the lumbar vertebrae. The resulting T‑score is determined by comparing the investigated person’s bone density values with those of an average young adult.

### Laboratory investigation

At the osteology outpatient department, the patients’ medical histories were recorded in detail and the patients underwent a clinical investigation including blood sampling, which was preferably performed from a cubital vein or another arm or hand vein (under the shortest possible period of congestion) by the doctor’s receptionist. The diseased persons had not fasted. Besides, blood sampling was not performed at a specific time but at the appointment in the outpatient department. The analysis was performed immediately in the medical laboratory of the hospital. The laboratory parameters for this investigation are described in the “Results” section.

### Non-pharmacological treatment of OP

#### Calcium and vitamin D

All patients were given calcium and vitamin D substitution as basic OP treatment. The aim of the treatment was to achieve a serum 25-hydroxyvitamin D level in excess of 50 nmol/l.

### Pharmacological OP treatment

#### Bisphosphonates

We used the following:

- Alendronate 10 mg daily or 70 mg a week orally
- Zolendronic acid 5 mg intravenously once a year in persons with a recent fracture and mild trauma, and long-term systemic glucocorticoid treatment in both sexes
- Ibandronate 3 mg as an intravenous injection every 3 months [5]

#### Denosumab

Given appropriate indications, the monoclonal antibody was administered as a subcutaneous injection at a dose of 60 mg every 6 months [5].

#### Teriparatide

Given appropriate indications, the osteoblast activator was administered as a subcutaneous injection at a daily dose of 20 µg for 24 months [5].

### Statistics

The collected data were analyzed with the statistical software package SPSS, Version 23.0 (SPSS Inc., Armonk, NY, USA). Quantitative variables were described in means (M), standard deviation (SD), and the number of available observations (*n*), as well as the interval M ± SD. For non-parametric tests the data were expressed in medians and the corresponding 1st and 3rd quartiles (Q1–Q3). For qualitative variables the individual degrees of severity as well as the absolute and percentage frequencies were given.

For group comparisons we used the Mann-Whitney U test. Selections were based on the result of the Shapiro-Wilk test for normal distribution. The Wilcoxon test was used for dependent group comparisons. Furthermore, correlation analyses were performed under consideration of scale dependencies. The probability of the non-occurrence of a VF under DT over time was expressed on a Kaplan-Meier curve. To compare the probability of the occurrence of VFs we used the log rank test. Furthermore, a Cox regression analysis was used to investigate the impact of independent factors on the occurrence of VFs. Variables that yielded a *p*-value < 0.05 on univariate analysis were introduced into the multivariate models. The probabilities of occurrence were depicted with the hazard ratio. All *p*-values were the result of two-sided statistical tests; the level of significance was set to *p* < 0.05.

## Results

### Basic characteristics of the study population

**Fig. 1:**
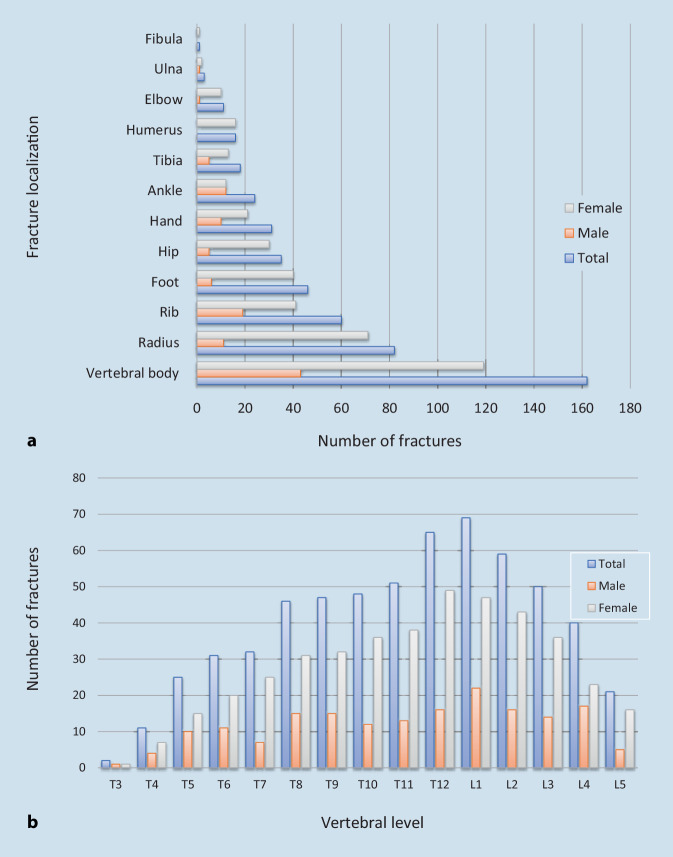
**a** Numbers of peripheral fractures in relation to gender and fracture location; distal radial fractures were the most common fracture in women and rib fractures were the most common in men. The distal radial fracture was the leading cause of peripheral fractures (27.3%) in the overall study population (*n* = 346). **b** VFs in relation to gender and vertebral fracture location; fractures were frequent at the thoracolumbar junction independent of gender; no VFs were observed above the thoracic vertebra Th3. *VFs* Vertebral fractures

A total of 346 patients participated in this clinical investigation. The mean age at the start of the study was 66.9 ± 10.7 years. The mean period of observation was 1384 days (range 59–3681 days, median 1372 days). The 346 patients consisted of 276 women (79.8%) and 70 men (20.2%). The age distribution revealed no significant differences between the sexes (*p* > 0.05). The BMI of men was significantly higher than that of women (*p* = 0.001). The basal metabolic rate was estimated using the Harris-Benedict formula and was also significantly higher in men (median 1585 kcal) than in women (median 1434 kcal, *p* < 0.001). In the total population, 61.3% experienced peripheral fractures. Radial (38.7%), rib (28.3%) and foot fractures (21.7%) were the most frequent types (Fig. 1a). Only 46.8% of the patients experienced a VF. Men (61.4%) had VFs significantly more often than women (43.1%, *p* = 0.006). At a reduced bone density (median −3.2 SD) we found numerous fusion fractures in the thoracic and thoracolumbar region (Fig. 1b). A group comparison of pain levels revealed a significant difference between men and women (*p* = 0.05).

The present patient population consisted mainly of older patients, partly with multimorbid conditions. Arterial hypertension was registered in 157 patients (45.4%), while 85 patients (24.6%) had diabetes mellitus. Significantly more men than women had coronary heart disease (men 15.7% vs. women 6.2%, *p* = 0.009). In all 65 patients (18.8%) had a malignant disease to start with; the diagnosis was significantly more frequent in men (27.1%) than in women (16.7%), 11 men (15.7%) and 13 women (4.7%) reported daily consumption of alcohol (*p* = 0.001).

Dividing OP patients into BDI und BDD groups revealed the following group differences: VFs were significantly more numerous in the BDD group (64%) (*p* < 0.001) than in the BDI (35.3%) group. Simultaneously, patients in the latter group reported a higher level of pain (*p* = 0.026).

Regarding the division of OP patients into those with and without VFs, we observed a significant age difference. Patients with VFs were older than those with no VFs (median age 72.8 years vs. 65.0 years; *p* < 0.001). Furthermore, men were significantly more numerous in the VF group than in the no VF group (27.5% vs. 14.5%; *p* = 0.003). The number of patients with marked thoracic kyphosis was significantly higher in the VF group than in the no VF group (46.4% vs. 23.8%; *p* < 0.001).

The OP patients with VFs had a significantly shorter pain-free walking (*p* = 0.004) and standing time (*p* = 0.005). Furthermore, they achieved fewer points on the SPPB (VFs 8.0 points vs. no VFs 9.0 points; *p* 0.012). Significantly fewer patients with VFs were able to lift and carry objects in their daily lives without pain (VFs 90.2% vs. no VFs 95.9%; *p* = 0.036). Furthermore, patients with VFs suffered more frequently from depression (VFs 19.0% vs. no VFs 9.8%; *p* = 0.015). Table 1 provides a summary of the patients’ medical histories, and Table 2 summarizes laboratory parameters at baseline (T0).

**Table 1:** Basic characteristics of the study population

Medical history	Overall group *n* = 346	No VFs under DT *n* = 193	VFs under DT *n* = 153	*p*-value	BDD under DT *n* = 139	BDI under DT *n* = 207	*p*-value
Follow-up (d). M ± SD	1384 ± 728	1521 ± 722	1212 ± 700	–	1333 ± 730	1419 ± 726	–
Follow-up (d) (min.–max.)	59–3681	59–3681	92–3569	–	92–3681	59–3569	–
Age (years)	68.5 (59.6–75.3)	65.0 (58.7–73.7)	72.8 (62.8–76.9)	**<** **0.001**^M^	67.8 (59.9–74.3)	69.0 (59.5–76.1)	0.563^M^
Gender (m/w)	70/276 (20.2%/79.8%)	28/165 (14.5%/85.5%)	42/111 (27.5%/72.5%)	**0.003**^C^	29/110 (20.9%/79.1%)	41/166 (19.8%/80.2%)	0.810^C^
Body mass index (kg/m^2^)	25.0 (23.0–28.0)	24.4 (22.5–27.0)	26.1 (23.2–29.3)	**<** **0.001**^M^	25.6 (23.0–28.0)	24.9 (22.9–28.0)	0.390^M^
Abdominal girth (cm)	98.0 (96.8–100.0)	98.0 (95.0–98.0)	98.0 (98.0–102.0)	**0.008**^M^	98.0 (97.0–101.0)	98.0 (96.0–98.0)	0.441^M^
Basal metabolic rate (kcal)	1435 (1339–1459)	1435 (1333–1445)	1435 (1356–1488)	0.083^M^	1435 (1336–1482)	1435 (1339–1445)	0.626^M^
*Drug therapy for OP*							
Bisphosphonates	195 (56.4%)	126 (65.3%)	69 (45.1%)	–	75 (54.0%)	120 (58.0%)	–
Teriparatide	8 (2.3%)	6 (3.1%)	2 (1.3%)		3 (2.2%)	5 (2.4%)	
Denosumab	26 (7.5%)	15 (7.8%)	11 (7.2%)		11 (7.9%)	15 (7.2%)	
Height difference (cm)	3.7 (1.8–6.0)	3.0 (1.4–4.5)	4.7 (2.0–8.0)	**0.001**^M^	3.9 (1.7–6.5)	3.5 (1.8–5.5)	0.367^M^
Peripheral fractures	212 (61.3%)	115 (59.6%)	97 (63.4%)	0.470^C^	80 (57.6%)	132 (63.8%)	0.245^C^
Vertebral fractures	162 (46.8%)	9 (4.7%)	153 (100%)	**<** **0.001**^C^	89 (64.0%)	73 (35.3%)	**<** **0.001**^C^
Fir tree phenomenon	62 (17.9%)	14 (7.3%)	48 (31.4%)	**<** **0.001**^C^	29 (20.9%)	33 (15.9%)	0.242^C^
Marked thoracic kyphosis	117 (33.8%)	46 (23.8%)	71 (46.4%)	**<** **0.001**^C^	50 (36.0%)	67 (32.4%)	0.487^C^
Pain (NRS 0–10)	0 (0–4)	0 (0–4)	0 (0–4.5)	0.870^M^	0 (0–5)	0 (0–2)	**0.026**^M^
Pain-free walking time (min)	30 (20–60)	60 (30–120)	30 (20–60)	**0.004**^M^	30 (20–90)	30 (20–60)	0.742^M^
Pain-free standing time (min)	30 (15–30)	30 (30–30)	30 (10–30)	**0.005**^M^	30 (15–30)	30 (15–30)	0.864^M^
SPPB (points)	8.0 (4.0–12.0)	9.0 (4.5–12.0)	8 (1–12)	**0.012**^M^	8 (4–12)	8 (4–12)	0.802^M^
Regular intake of calcium (mg)	700 (450–1000)	800 (450–1000)	700 (450–1000)	0.890^M^	700 (450–950)	800 (500–1000)	0.230^M^
Cachectic phases of life	51 (14.7%)	20 (10.4%)	31 (20.3%)	**0.010**^C^	12 (8.6%)	39 (18.8%)	**0.009**^C^
Daily consumption of alcohol	24 (6.9%)	10 (5.2%)	14 (9.2%)	0.149^C^	9 (6.5%)	15 (7.2%)	0.782^C^
Smoking	18 (5.2%)	9 (4.7%)	9 (5.9%)	0.612^C^	8 (5.8%)	10 (4.8%)	0.704^C^
Lifting and carrying objects regularly	323 (93.4%)	185 (95.9%)	138 (90.2%)	**0.036**^C^	129 (92.8%)	194 (93.7%)	0.738^C^
*Comorbidities*							
Osteoarthritis of the hip or knee	75 (21.7%)	37 (19.2%)	38 (24.8%)	0.204^C^	37 (26.6%)	38 (18.4%)	0.067^C^
Fibromyalgia syndrome	6 (1.7%)	3 (1.6%)	3 (2.0%)	0.774^C^	0 (0%)	6 (2.9%)	**0.043**^C^
Osteoarthritis of the spine	7 (2.0%)	6 (3.1%)	1 (0.7%)	0.107^C^	3 (2.2%)	4 (1.9%)	0.884^C^
Rheumatoid arthritis	26 (7.5%)	13 (6.7%)	13 (8.5%)	0.537^C^	13 (9.4%)	13 (6.3%)	0.288^C^
Chronic regional pain syndrome	3 (0.9%)	3 (1.6%)	0 (0%)	0.121^C^	0 (0%)	3 (1.4%)	0.154^C^
Psoriatic arthritis	4 (1.2%)	2 (1.0%)	2 (1.3%)	0.915^C^	0 (0%)	4 (1.9%)	0.099^C^
Arterial hypertension	157 (45.4%)	85 (44%)	72 (47.1%)	0.576^C^	65 (46.8%)	92 (44.4%)	0.671^C^
Diabetes mellitus	85 (24.6%)	45 (23.3%)	40 (26.1%)	0.544^C^	37 (26.6%)	48 (23.2%)	0.467^C^
Hyperlipoproteinemia	60 (17.3%)	38 (19.7%)	22 (14.4%)	0.195^C^	27 (19.4%)	33 (15.9%)	0.402^C^
Hyperuricemia	20 (5.8%)	11 (5.7%)	9 (5.9%)	0.942^C^	9 (6.5%)	11 (5.3%)	0.650^C^
Renal failure	32 (9.2%)	16 (8.3%)	16 (10.5%)	0.489^C^	15 (10.8%)	17 (8.2%)	0.417^C^
Hyperparathyroidism	31 (9.0%)	19 (9.8%)	12 (7.8%)	0.517^C^	13 (9.4%)	18 (8.7%)	0.834^C^
Cerebral stroke	7 (2.0%)	3 (1.6%)	4 (2.6%)	0.487^C^	3 (2.2%)	4 (1.9%)	0.884^C^
Temporal arteritis	2 (0.6%)	1 (0.5%)	1 (0.7%)	0.869^C^	1 (0.7%)	1 (0.7%)	0.776^C^
Coronary heart disease	28 (8.1%)	12 (6.2%)	16 (10.5%)	0.151^C^	13 (9.4%)	15 (7.2%)	0.481^C^
Atrial fibrillation	25 (7.2%)	12 (6.2%)	13 (8.5%)	0.416^C^	11 (7.9%)	14 (6.8%)	0.685^C^
Hypothyroidism	19 (5.5%)	11 (5.7%)	8 (5.2%)	0.849^C^	10 (7.2%)	9 (4.3%)	0.255^C^
Hyperthyroidism	9 (2.6%)	7 (3.6%)	2 (1.3%)	0.178^C^	2 (1.4%)	7 (3.4%)	0.266^C^
Bronchial asthma	16 (4.6%)	9 (4.7%)	7 (4.6%)	0.969^C^	7 (5.0%)	9 (4.3%)	0.765^C^
Chronic obstructive pulmonary disease	17 (4.9%)	9 (4.7%)	8 (5.2%)	0.809^C^	7 (5.0%)	10 (4.8%)	0.931^C^
Peripheral arterial occlusive disease	2 (0.6%)	1 (0.5%)	1 (0.7%)	0.869^C^	1 (0.7%)	1 (0.5%)	0.776^C^
Hepatitis	3 (0.9%)	1 (0.5%)	2 (1.3%)	0.432^C^	2 (1.4%)	1 (0.5%)	0.347^C^
Irritable bowel syndrome	3 (0.9%)	3 (1.6%)	0 (0%)	0.121^C^	1 (0.7%)	2 (1.0%)	0.808^C^
Gastroesophageal reflux disease	9 (2.6%)	6 (3.1%)	3 (2.0%)	0.505^C^	4 (2.9%)	5 (2.4%)	0.791^C^
Malignant disease	65 (18.8%)	38 (19.7%)	27 (17.6%)	0.629^C^	22 (15.8%)	43 (20.8%)	0.248^C^
Depression	48 (13.9%)	19 (9.8%)	29 (19.0%)	**0.015**^C^	10 (13.7%)	29 (14.0%)	0.928^C^
Epilepsy	8 (2.3%)	5 (2.6%)	3 (2.0%)	0.699^C^	3 (2.2%)	5 (2.4%)	0.876^C^

Data are expressed in numbers (*n*) and percentages (%) of the group, mean value ± standard deviation (M ± SD) and medians with the 1st and the 3rd quartiles (QI–QIII), χ^2^-test^C^, Mann-Whitney U test^M^. Significant results are highlighted in *bold print*.

*min.–max.* minimum–maximum, *d* days, *BDD* bone density decrease, *BDI* bone density increase, *DOP* drug treatment for osteoporosis, *VFs* vertebral fractures, *LS* lumbar spine, *NRS* numeric rating scale, *SPPB* short physical performance battery

**Table 2:** Laboratory parameters

Parameter	Men	Women
Erythrocytes (10^12^/l)	4.72 ± 0.52	4.46 ± 0.45
Leukocytes (10^9^/l)	7.30 ± 2.83	6.98 ± 2.20
Thrombocytes (10^9^/l)	232.93 ± 83.15	251.07 ± 63.83
Hemoglobin (mmol/l)	8.95 ± 0.95	8.42 ± 0.87
Hematocrit (%)	0.42 ± 0.04	0.40 ± 0.03
MCH (fmol)	1.90 ± 0.13	1.89 ± 0.10
MCV (fl)	90.47 ± 4.71	90.44 ± 4.97
Sodium (mmol/l)	139.1 ± 2.4	138.5 ± 2.8
Potassium (mmol/l)	4.5 ± 0.7	4.5 ± 0.5
Creatinine (µmol/l)	92.2 ± 35.5	75.3 ± 46.5
eGFR (ml/min)	83.1 ± 21.7	84.1 ± 30.4
*Bone metabolism*		
Ostase (µg/l)	13.5 ± 5.7	14.3 ± 9.1
TRAP 5b (U/l)	3.38 ± 1.34	3.92 ± 1.56
Parathyroid hormone (pmol/l)	6.2 ± 2.7	5.7 ± 2.8
Vitamin D 25-OH (nmol/l)	63.3 ± 23.5	68.1 ± 28.9
Calcium (mmol/l)	2.4 ± 0.2	2.5 ± 0.2
Phosphate (mmol/l)	1.08 ± 0.17	1.16 ± 0.17

Data are expressed as means ± standard deviation (M ±SD).

The numbers are in SI units

### Procedural results under DT

#### BMD

**Fig. 2:**
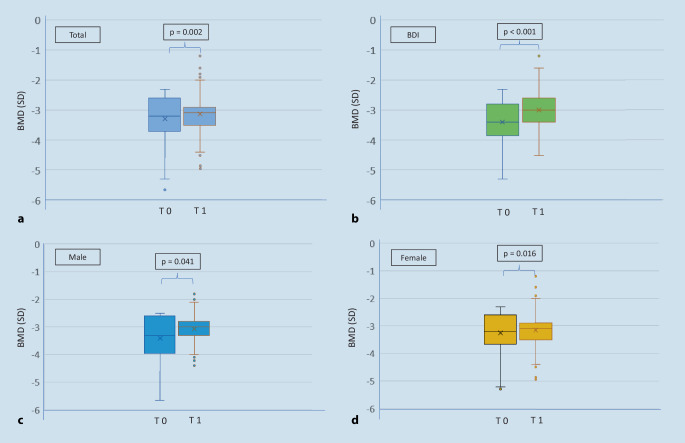
Comparison of bone density in the overall group and relevant subgroups on dual X‑ray absorptiometry (T-score in SD) at baseline (T 0) vs. follow-up (T 1). The mean duration of follow-up was 1449 ± 635 days. Bone density increased significantly in the overall group (**a**) as well as in subgroups (**b** BDI, **c** male, **d** female) under DT. *BMD* bone mineral density, *SD* standard deviation, *BDI* bone density increase, *DT* drug treatment for osteoporosis

At baseline the overall group had a median BMD of −3.2 SD. This value rose significantly to −3.1 SD (*p* = 0.002) in the follow-up period (Table 3). Patients with VFs had a significantly lower BMD than patients without VFs (*p* = 0.009). Patients with a very low BMD at baseline developed a significantly higher increase than patients with higher BMD (BDI −3.4 SD vs. BDD −3.0 SD; *p* < 0.001). The T‑score increased in men (*p* = 0.041), women (*p* = 0.016), BDI (*p* < 0.001) (Fig. 2), and VFs patients (*p* = 0.002) (Table 4); however, patients without VFs revealed no significant difference (*p* > 0.05).

**Table 3:** Comparison of bone density and hand grip strength between groups

	Overall *n* = 346	Men *n* = 70	Women *n* = 276	*p*-value^M^	No VFs *n* = 193	VFs *n* = 153	*p*-value^M^	BDD *n* = 139	BDI *n* = 207	*p*-value^M^
*Baseline*										
BMD (T-score at LS)	−3.2 (−3.7 to −2.6)	−3.3 (−4.0 to −2.6)	−3.2 (−3.7 to −2.6)	0.186	−3.1 (−3.6 to −2.5)	−3.3 (−3.9 to −2.7)	**0.009**	−3.0 (−3.4 to −2.5)	−3.4 (−3.8 to −2.8)	**<** **0.001**^M^
HGS (kg)	26.0 (21.0–32.0)	42.0 (35.5–50.0)	24.0 (20.0–28.0)	**<** **0.001**	26.0 (22.0–32.0)	26.0 (20.0–33.0)	0.514	26.0 (22.0–33.0)	26.0 (21.0–32.0)	0.433
*Follow-up*										
BMD (T-score at LS)	−3.1 (−3.5 to −2.9)	−3.0 (−3.3 to −2.8)	−3.1 (−3.5 to −2.9)	0.265	−3.1 (−3.3 to −2.9)	−3.1 (−3.6 to −2.9)	0.118	−3.2 (−3.6 to −3.0)	−3.0 (−3.4 to −2.6)	**<** **0.001**
HGS (kg)	24.0 (20.0–31.0)	36.0 (28.0–46.0)	23.0 (20.0–28.0)	**<** **0.001**	25.0 (20.5–30.5)	24.0 (20.0–32.0)	0.719	26.0 (20.0–31.0)	24.0 (20.0–31.5)	0.473

Data are expressed as medians with the 1st and 3rd quartiles (QI–QIII), Mann-Whitney U test^M^

*BMD* bone mineral density, *HGS* hand grip strength, *BDD* bone density decrease, *BDI* bone density increase, *VFs* Vertebral fractures, *LS* lumbar spine

**Table 4:** Comparison of bone density and hand grip strength within groups

	BMD (T-score at the LS in SD)			HGS (kg)		
	Baseline	Follow-up	*p*-value^W^	Baseline	Follow-up	*p*-value^W^
Overall	−3.2 (−3.7 to −2.6)	−3.1 (−3.5 to −2.9)	**0.002**	26.0 (21.0–32.0)	24.0 (20.0–31.0)	**<** **0.001**
Men	−3.3 (−4.0 to −2.6)	−3.0 (−3.3 to −2.8)	**0.041**	42.0 (35.5–50.0)	36.0 (28.0–46.0)	**<** **0.001**
Women	−3.2 (−3.7 to −2.6)	−3.1 (−3.5 to −2.9)	**0.016**	24.0 (20.0–28.0)	23.0 (20.0–28.0)	**<** **0.001**
BDD	−3.0 (−3.4 to −2.5)	−3.2 (−3.6 to −3.0)	**<** **0.001**	26.0 (22.0–33.0)	26.0 (20.0–31.0)	**<** **0.001**
BDI	−3.4 (−3.8 to −2.8)	−3.0 (−3.4 to −2.6)	**<** **0.001**	26.0 (21.0–32.0)	24.0 (20.0–31.5)	**<** **0.001**
No VFs	−3.1 (−3.6 to −2.5)	−3.1 (−3.3 to −2.9)	0.177	26.0 (22.0–32.0)	25.0 (20.5–30.5)	**<** **0.001**
VFs	−3.3 (−3.9 to −2.7)	−3.1 (−3.6 to −2.9)	**0.002**	26.0 (20.0–33.0)	24.0 (20.0–32.0)	**<** **0.001**

Data are expressed as medians with the 1st and 3rd quartiles (QI–QIII), Wilcoxon test^W^

*BMD* bone mineral density, *SD* standard deviation, *BDD* bone density decrease, *BDI* bone density increase, *HGS* hand grip strength, *VFs* vertebral fractures, *LS* lumbar spine

#### HGS

On the HGS test, the study participants achieved a median value of 26.0 kg with the dominant hand. The test yielded values between 4 kg and 80 kg. Men achieved a median value of 42 kg on the HGS test at baseline. Women, on the other hand, achieved 24 kg at this time point (*p* < 0.001). Independent of changes in the different groups no significant difference was noted between the groups (*p* > 0.05; Table 3).

**Fig. 3:**
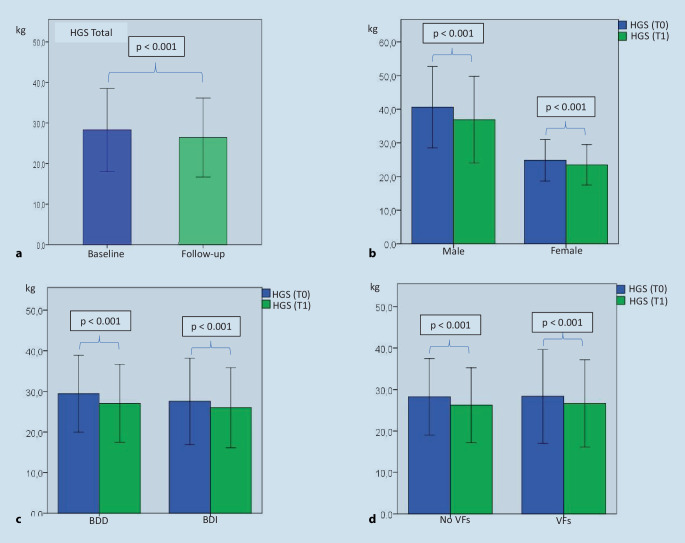
Development of hand grip strength (HGS) in the dominant hand; **a** comparisons within the entire group; baseline (T0) vs. follow-up (T1); **b** gender-dependent comparison of HGS T0 vs. T1; **c** comparison of groups with and without a bone density increase T0 vs. T1; **d** comparison of groups with and without new VFs T0 vs. T1. *HGS* hand grip strength, *BDD* bone density decrease, *BDI* bone density increase, *No VFs* no vertebral fractures, *VFs* vertebral fractures

The HGS in the overall group was significantly reduced at the follow-up investigation (T0 26.0 kg vs. T1 24.0 kg; *p* < 0.001, Fig. 3). Tables 3 and 4 provide a summary of the measured values.

Significant correlations were registered between HGS in the dominant hand and the age of OP patients (r = −0.337, *p* < 0.001), HGS and BMI (r = 0.153, *p* = 0.004), HGS and the SPPB (r = 0.123, *p* = 0.023), HGS and the NRS (r = −0.118, *p* = 0.029), and HGS and TRAP (tartrate-resistant acid phosphatase) 5b (r = −0.142, *p* = 0.008) (Table 5).

**Table 5:** Spearman’s correlation coefficients between hand grip strength and other medical history-based and laboratory parameters

	HGS	Age	BMI	NRS	SPPB	TRAP 5b
HGS (kg)	–	–	–	–	–	–
Age (years)	−0.337***	–	–	–	–	–
BMI (kg/m^2^)	0.153*	0.071	–	–	–	–
NRS (0–10)	−0.118*	−0.051	−0.058	–	–	–
SPPB (*n*)	0.123*	−0.167**	−0.245***	0.323***	–	–
TRAP 5b (U/l)	−0.142**	0.044	−0.236***	0.127*	0.130*	–

*HGS* hand grip strength, *BMI* body mass index, *NRS* numeric rating scale, *SPPB* Short physical performance battery, *TRAP 5b* tartrate-resistant acid phosphatase

*p < 0.05, **p < 0.01, ***p < 0.001

### Kaplan-Meier and multivariate regression analysis

**Fig. 4:**
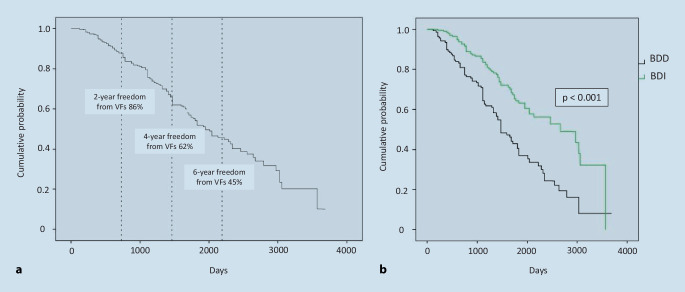
Kaplan-Meier curve of the probability of no vertebral fractures (VFs). Exemplary presentation after 2, 4 and 6 years; the median time until the occurrence of a VF was 1943 days for the overall group (95% CI 1649.4–2236.6 days). **a** Kaplan-Meier curve of the group comparison of BDI vs. BDD. The success of treatment was defined as an improvement of the T‑score on DXA. The median time until the occurrence of a VF was 1461 days in the BDD group (95% CI 1246.5–1675.5 days) and 2652 days in the BDI group (95% CI 1825.2–3478.8 days, *p* < 0.001). *BDD* bone density decreas, *BDI* bone density increase

The Kaplan-Meier analysis permitted a calculation of freedom from VFs (Fig. 4). The probability of achieving a specific time point without VFs was determined by dividing the number of patients exposed to risks defined according to the time point of investigation by the number of patients at risk before the time point. Two years after the start of DT, 86% of patients were free of a VF. After 4 years 62%, and after 6 years 45% of the patients were not affected by a VF (Fig. 4a). The median time until the occurrence of a VF was 1943 days in the overall group (95% CI 1649.4–2236.6).

The median time until the occurrence of a VF was 1461 days in patients without an increase in BMD (95% CI 1246.5–1675.5) and much longer, 2652 days, in patients with an increase in BMD (CI 1825.2–3478.8 days; *p* < 0.001, Fig. 4b).

In a Cox multivariate analysis, independent predictors of a VF were determined for the entire group. In addition to a missing of BDI (*p* < 0.001) the predictors included age (*p* = 0.010), gender (*p* = 0.002) and NRS (*p* < 0.001). The highest hazard ratio was noted for gender (Table 6).

**Table 6:** Predictors of the probability of a vertebral fracture

			95% CI of the hazard ratio	
Parameter	*p*-value	Hazard ratio	Lower	Upper
BDI (yes/no)	*<* *0.001*	0.461	0.334	0.637
Age (years)	*0.010*	1.022	1.005	1.039
Gender (male/female)	*0.002*	1.769	1.232	2.540
Numeric rating scale (0–10)	*<* *0.001*	1.125	1.062	1.192

*BDI* bone density increase, *CI* confidence interval

## Discussion

The present investigation is the first to provide comprehensive data on the effects of DT over a period of 10 years on performance parameters and bone density in a real-world setting. Bone density could be significantly increased in the overall group, whereas HGS remained unchanged or even worsened. While the reasons for these changes are manifold, we presume that DT had an impact on bone density.

The potential value of BMD in predicting the VF reduction rate with antiresorptive treatment has been reported in previous clinical studies [10]. A recently published meta-regression analysis showed that an increase in overall BMD at the hips by 2% may be able to reduce the VF risk by 28% [4].

In the present investigation, patients without VFs as well as patients with a BDI took more bisphosphonates. Bisphosphonates may cause an accumulation of microdamage and thus impair the healing of stress fractures [33]; however, we did not observe this in our investigation.

In the present investigation, all participants were advised to take 20,000 IU vitamin D3 per week. The aim of the treatment was to achieve a serum 25-hydroxyvitamin D level in excess of 50 nmol/l.

The overall significant reduction of HGS in all groups was remarkable. The present results indirectly support the association between HGS and BMD. HGS was correlated with NRS. The latter, in turn, was correlated with the success of treatment, defined as an improvement of the T‑score at the LS in OP patients.

Dixon et al. [12] investigated 1265 men and 1380 women aged over 50 years and concluded that a low HGS is associated with a low BMD of the spine as well as the hips and an elevated risk of VFs. After completing their investigation of 1039 women aged on average 73 years, Eguchi et al. [15] concluded that the loss of muscle mass in the lower extremities and HGS are closely associated with the occurrence of VFs.

In a large German study, reference values were published for HGS in women and men of different age groups in relation to body height. [32]. In addition to patients of similar ethnic origin as in our study, the authors used a dynamometer of the same brand to perform and classify the measurements. Women aged 65–69 years with a body height of 1.60–1.64m achieved a mean HGS of 27.5 kg in the dominant hand. In this constellation, an HGS of 22.3 kg is viewed as the risk threshold [32]. In the present study, women (average 67.4 years and 1.60m) had an average HGS of 24.4 kg in the dominant hand, which was above the mean value for their age. For men the reference value of HGS is 43.6 kg at a risk threshold of 36.3 kg. Men (65.1 years, 1.73m) achieved an average HGS of 41.4 kg with the dominant hand and were thus somewhat below the mean value for their age, but markedly above the risk threshold. The scores were not below the cut-offs for sarcopenia with reference to gender and groups. The general reduction of HGS across groups creates the impression that the effect on specificity is attributable to strength. In other words, only those patients who do exercises for muscle strength actually increase the muscle strength. Some of the participants attended the muscle strengthening program regularly, whereas others swam or rode a bicycle. The results of our study show that in addition to DT, consistent additional muscle exercises are meaningful.

The present investigation showed a negative correlation between HGS and age. HGS reduces significantly with advancing age, regardless of whether a patient has OP or not. It should be noted that HGS was correlated with SPPB especially with the in the SPPB included CRT. This leads to the presumed conclusion that an increase in HGS permits the trunk to be balanced more easily; however, comparative studies are currently not available. With respect to previous VFs and physical performance parameters, the Makarova et al. study [26] showed that VFs in OP are associated with a significant reduction in the strength of all body muscles, especially the deep stabilizers of the spine.

Early detection of VFs is important because VFs constitute a markedly higher risk of future osteoporotic fractures. In the present study, patients with VFs suffered more often from depression, had kyphotic changes in the TS and a corresponding fir tree phenomenon more frequently. We found that independent factors influencing the occurrence of a VF included, in addition to age and gender, the improvement of the T‑score. The severity of back pain determined on the NRS is also a predictor of future VFs. As OP is commonly a silent clinical condition and is not diagnosed early, it is usually identified late or not at all [22].

## Limitations

The so-called real-world design and the non-blinding of patients as well as investigators are limitations of the present study. Furthermore, the different OP drugs, as well as their changes during treatment might have influenced the validity of the conclusions. In oral DT, patient compliance should be reviewed critically.

The total mean investigation period of 4 years was sufficient to draw conclusions about the long-term effect of treatment, but at the time of follow-up complete data were available for a mere 62% of patients, which was attributable to the real-world character of the current study. In future studies we intend to improve the evidence by performing an ongoing evaluation with long-term compliance on the part of patients.

## Conclusion

- Guideline-based DT improved bone density, but there is no association with HGS.
- The time until the occurrence of a VF was shorter in a subgroup of patients without a bone density increase despite taking DT.
- Age, gender, missing bone density increase and back pain were independent predictors of new VFs.
- DT alone appears to be insufficient for the prevention of osteosarcopenia.
- A personal fitness program aligned to the individual patient and early establishment of a muscle exercise program appear to be meaningful along with guideline-based DT for OP.

## Abbreviations

BDD: Bone density decrease
BDI: Bone density increase
BMD: Bone mineral density
BMI: Body mass index
CRT: Chair rising test
DT: Drug treatment
DXA: Dual X‑ray absorptiometry
HGS: Hand grip strength
LS: Lumbar spine
M: Mean value
NRS: Numeric rating scale
OP: Osteoporosis
SD: Standard deviation
SPPB: Short physical performance battery
TDS: Tandem stance
TG: Tandem gait
TS: Thoracic spine
VFs: Vertebral fractures

## Declarations

All procedures performed in studies involving human participants or on human tissue were in accordance with the ethical standards of the institutional and/or national research committee and with the 1975 Helsinki declaration and its later amendments or comparable ethical standards. Informed consent was obtained from all individual participants included in the study.

## References

[1] Bartl R, Bartl C, Mutschler W (2003). Diagnostik und Therapie der Osteoporose. Strategie für eine effiziente Prävention von Folgefrakturen. Unfallchirurg.

[2] Basler H-D (2011). Akutschmerztherapie in Pädiatrie und Geriatrie – Schmerzmessung: Welche Schmerzskala bei welchen Patienten?. Anasthesiol Intensivmed Notfallmed Schmerzther.

[3] Bässgen K, Westphal T, Haar P, Kundt G, Mittlmeier T, Schober H-C (2013). Population-based prospective study on the incidence of osteoporosis-associated fractures in a German population of 200,413 inhabitants. J Public Health (Oxf).

[4] Bouxsein ML, Eastell R, Lui L-Y, Wu LA, de Papp AE, Grauer A, Marin F, Cauley JA, Bauer DC, Black DM (2019). Change in bone density and reduction in fracture risk: a meta-regression of published trials. J Bone Miner Res.

[5] Compston J, Cooper A, Cooper C, Gittoes N, Gregson C, Harvey N, Hope S, Kanis JA, McCloskey EV, Poole KES, Reid DM, Selby P, Thompson F, Thurston A, Vine N (2017). UK clinical guideline for the prevention and treatment of osteoporosis. Arch Osteoporos.

[6] Cooper R, Kuh D, Hardy R (2010). Objectively measured physical capability levels and mortality: systematic review and meta-analysis. BMJ.

[7] Cooper C, Fielding R, Visser M, van Loon LJ, Rolland Y, Orwoll E, Reid K, Boonen S, Dere W, Epstein S, Mitlak B, Tsouderos Y, Sayer AA, Rizzoli R, Reginster JY, Kanis JA (2013). Tools in the assessment of sarcopenia. Calcif Tissue Int.

[8] Cruz-Jentoft AJ, Sayer AA (2019). Sarcopenia. Lancet.

[9] Cruz-Jentoft AJ, Bahat G, Bauer J, Boirie Y, Bruyère O, Cederholm T, Cooper C, Landi F, Rolland Y, Sayer AA, Schneider SM, Sieber CC, Topinkova E, Vandewoude M, Visser M, Zamboni M (2019). Sarcopenia: revised European consensus on definition and diagnosis. Age Ageing.

[10] Cummings SR, Karpf DB, Harris F, Genant HK, Ensrud K, LaCroix AZ, Black DM (2002). Improvement in spine bone density and reduction in risk of vertebral fractures during treatment with antiresorptive drugs. Am J Med.

[11] Daly RM, Ahlborg HG, Ringsberg K, Gardsell P, Sernbo I, Karlsson MK (2008). Association between changes in habitual physical activity and changes in bone density, muscle strength, and functional performance in elderly men and women. J Am Geriatr Soc.

[12] Dixon WG, Lunt M, Pye SR, Reeve J, Felsenberg D, Silman AJ, O’Neill TW (2005). Low grip strength is associated with bone mineral density and vertebral fracture in women. Rheumatology.

[13] Dodds RM, Syddall HE, Cooper R, Benzeval M, Deary IJ, Dennison EM, Der G, Gale CR, Inskip HM, Jagger C, Kirkwood TB, Lawlor DA, Robinson SM, Starr JM, Steptoe A, Tilling K, Kuh D, Cooper C, Sayer AA (2014). Grip strength across the life course: normative data from twelve British studies. PLoS ONE.

[14] Edwards MH, Gregson CL, Patel HP, Jameson KA, Harvey NC, Sayer AA, Dennison EM, Cooper C (2013). Muscle size, strength, and physical performance and their associations with bone structure in the Hertfordshire cohort study. J Bone Miner Res.

[15] Eguchi Y, Toyoguchi T, Orita S, Shimazu K, Inage K, Fujimoto K, Suzuki M, Norimoto M, Umimura T, Shiga Y, Inoue M, Koda M, Furuya T, Maki S, Hirosawa N, Aoki Y, Nakamura J, Hagiwara S, Akazawa T, Takahashi H, Takahashi K, Shiko Y, Kawasaki Y, Ohtori S (2019). Reduced leg muscle mass and lower grip strength in women are associated with osteoporotic vertebral compression fractures. Arch Osteoporos.

[16] Fried LP, Tangen CM, Walston J, Newman AB, Hirsch C, Gottdiener J, Seeman T, Tracy R, Kop WJ, Burke G, McBurnie MA (2001). Frailty in older adults: evidence for a phenotype. J Gerontol A Biol Sci Med Sci.

[17] Guralnik JM, Simonsick EM, Ferrucci L, Glynn RJ, Berkman LF, Blazer DG, Scherr PA, Wallace RB (1994). A short physical performance battery assessing lower extremity function: association with self-reported disability and prediction of mortality and nursing home admission. J Gerontol.

[18] Guralnik JM, Ferrucci L, Pieper CF, Leveille SG, Markides KS, Ostir GV, Studenski S, Berkman LF, Wallace RB (2000). Lower extremity function and subsequent disability: consistency across studies, predictive models, and value of gait speed alone compared with the short physical performance battery. J Gerontol A Biol Sci Med Sci.

[19] Haidar SG, Kumar D, Bassi RS, Deshmukh SC (2004). Average versus maximum grip strength: which is more consistent?. J Hand Surg Br.

[20] Häussler B, Gothe H, Göl D, Glaeske G, Pientka L, Felsenberg D (2007). Epidemiology, treatment and costs of osteoporosis in Germany—the BoneEVA study. Osteoporos Int.

[21] Johnell O, Kanis JA (2006). An estimate of the worldwide prevalence and disability associated with osteoporotic fractures. Osteoporos Int.

[22] Kanis JA, Norton N, Harvey NC, Jacobson T, Johansson H, Lorentzon M, McCloskey EV, Willers C, Borgström F (2021). SCOPE 2021: a new scorecard for osteoporosis in Europe. Arch Osteoporos.

[23] Kendler DL, Bauer DC, Davison KS, Dian L, Hanley DA, Harris ST, McClung MR, Miller PD, Schousboe JT, Yuen CK, Lewiecki EM (2016). Vertebral fractures: clinical importance and management. Am J Med.

[24] Lang T, Cauley JA, Tylavsky F, Bauer D, Cummings S, Harris TB (2010). Computed tomographic measurements of thigh muscle cross-sectional area and attenuation coefficient predict hip fracture: the health, aging, and body composition study. J Bone Miner Res.

[25] Ling CHY, Taekema D, de Craen AJM, Gussekloo J, Westendorp RGJ, Maier AB (2010). Handgrip strength and mortality in the oldest old population: the Leiden 85-plus study. CMAJ.

[26] Makarova EV, Marchenkova LA, Eryomushkin MA, Styazkina EM, Chesnikova EI (2020). Balance and muscle strength tests in patients with osteoporotic vertebral fractures to develop tailored rehabilitation programs. Eur J Transl Myol.

[27] Martin-Ruiz C, von Zglinicki T (2014). Biomarkers of healthy ageing: expectations and validation. Proc Nutr Soc.

[28] Roberts HC, Denison HJ, Martin HJ, Patel HP, Syddall H, Cooper C, Sayer AA (2011). A review of the measurement of grip strength in clinical and epidemiological studies: towards a standardised approach. Age Ageing.

[29] Runge M (2009). Fünf Esslinger. Ein Bewegungsprogramm für Muskel-Knochen-Fitness.

[30] Sallakhi A, Andresen JR, Schröder G, Andresen R, Schober H-C (2021). Abnehmende Handkraft als Indikator für eine erhöhte Sterblichkeit bei Patienten unter alleiniger spezifischer Osteoporosetherapie. Osteologie.

[31] Stanghelle B, Bentzen H, Giangregorio L, Pripp AH, Bergland A (2018). Effect of a resistance and balance exercise programme for women with osteoporosis and vertebral fracture: study protocol for a randomized controlled trial. BMC Musculoskelet Disord.

[32] Steiber N (2016). Strong or weak handgrip? Normative reference values for the German population across the life course stratified by sex, age, and body height. PLoS ONE.

[33] Unnanuntana A, Saleh A, Mensah KA, Kleimeyer JP, Lane JM (2013). Atypical femoral fractures: what do we know about them?: AAOS exhibit selection. J Bone Joint Surg Am.

[34] Zanker J, Duque G (2019). Osteoporosis in older persons: old and new players. J Am Geriatr Soc.

